# Introducing Nafion for *In Situ* Desalting and Biofluid Profiling in Spray Mass Spectrometry

**DOI:** 10.3389/fchem.2021.807244

**Published:** 2022-01-25

**Authors:** Xiaowei Song, Mohammad Mofidfar, Richard N. Zare

**Affiliations:** ^1^ Department of Chemistry, Fudan University, Shanghai, China; ^2^ Department of Chemistry, Stanford University, Stanford, CA, United States

**Keywords:** Nafion, desalting, spray mass spectrometry, biofluid, metabolic profiling

## Abstract

We introduce Nafion into the ambient ionization technique of spray mass spectrometry to serve for *in situ* desalting and direct analysis of biological fluids. Nafion was coated onto the surface of the triangular spray tip as the cation exchange material. Because the sulfonic group from the Nafion membrane effectively exchanges their carried protons with inorganic salt ions (e.g., Na^+^ and K^+^), the analyte’s ionization efficiency can be significantly enhanced by reducing ion suppression. The desalting efficiency can reach 90% and the maximum tolerance of the absolute salt amount reaches 100 μmol. The mass spectral profile can also be simplified by removing the multiple adducted ion types from small-molecule drugs and metabolites ([M + Na]^+^ and [M + K]^+^), or multiply charged ions formed by proteins ([M + nNa]^n+^ and [M + nK]^n+^). Thus, the Nafion coating makes less ambiguous data interpretation collected from spray mass spectrometry for qualitative profiling or quantitative measurement of a target analyte.

## Introduction

Ambient ionization-based spray mass spectrometry (spray MS) is one of the most evolving directions in the mass spectrometry field owing to its remarkable advantages in pretreatment-free sampling and direct MS data collection under atmospheric conditions (Ma and Ouyang, 2016; Feider et al., 2019). It has been successfully used for clinical disease diagnosis and drug monitoring with various biological fluids as tested samples such as saliva (Song et al., 2020), blood or serum (Ma et al., 2015; Li et al., 2021), urine (Rossini et al., 2020), tears (Yao et al., 2020), and tissue (Yan et al., 2020). These biofluids contain a multitude of endogenous species associated with physiological functions including amino acids, carbohydrates, lipids, nucleotides, and proteins.

However, the matrix effect poses a harsh technical issue for the spray MS analysis of biological fluid samples (Vega et al., 2016). The high concentration of inorganic salts (e.g., NaCl and KCl) was one of the major factors contributing to the matrix effect. They will not only cause contamination to a mass spectrometer during the sample introduction process but also severely suppress the ionization efficiency of a target analyte within a sample. Therefore, the sensitivity becomes quite weak for those species that have low abundances or ionization efficiencies. In terms of those high abundance components, their mass spectra also become complicated by forming multiple types of adduct ions such as [M + H]^+^, [M + Na]^+^, and [M + K]^+^. This phenomenon can be particularly observed in the mass spectrometric analysis of nucleotides and proteins. The protons from nucleotide’s phosphoric acid group are prone to exchange with more than one sodium ion under the physiological pH around 7.4. The intensity of protein ions will also be greatly lowered because of highly dispersed adduct ion distribution.

For MS spray analysis, porous media is one of the major sample-loading substrates such as paper (Wang et al., 2010), sponge (Hecht et al., 2017), swab (Pruski et al., 2021), and wooden tip (Hu et al., 2018). These materials have a large contacting surface and fiber structure to hinder macromolecules or insoluble particles moving forward the MS inlet after mounting extraction solvent. However, these inorganic salts cannot be stopped from ionizing and adducting on the analytes in biofluids. Recently, the use of acetonitrile–water (9:1, v/v) cooperated with a Kimwipe paper was reported to effectively wash away salts from oligosaccharide samples for paper spray ionization (Wang et al., 2020; Chiu et al., 2021). Apart from the liquid solution washing strategy, hydrogel was also introduced for salts removal (Song et al., 2017). However, this method works better for hydrophobic drugs that can be well preserved after the desalting process.

There is also an online desalting method that uses alternating electrostatic field for first inducing cation salt ions away from the analyte and then switching the field polarity for triggering the electrospray process (Wei et al., 2019). It is mainly used in the target analysis of a limited number of interesting compounds from a simplified solvent system such as product and substrate in an enzymatic reaction or an organic reaction. This physical method can be well compatible with the nano-electrospray ionization (nESI) and needs a more sophisticated electronic setup to implement. A much easier online desalting method is still in urgent demand for conducting large-scale biological fluid profiling in a robust and universally applicable way.

**FIGURE 1:**
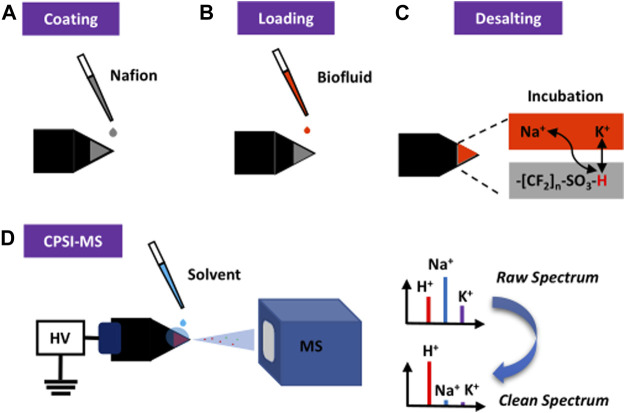
Principal and general procedure of Nafion-coating conductive polymer spray ionization mass spectrometry for direct analysis of biofluid. **(A)** Nafion solution is coated onto the surface of the conductive polymer tip. **(B)** After the solution is evaporated to form a thin membrane, the trace volume (1 μl) of diluted biofluid is directly loaded onto the membrane surface. **(C)** The Nafion desalting process is then initiated and lasted until the biofluid becomes a dried spot. **(D)** When another drop of solvent was added onto the dried biofluid spot, a high voltage triggers the start of CPSI-MS data acquisition.

Here, we report the use of Nafion, a sulfonated tetrafluoroethylene-based fluoropolymer used in fuel cells, to effectively remove inorganic salts for spray MS based on the cation exchange mechanism. As long as two decades ago, Nafion has been reported as the matrix-assisted laser desorption ionization (MALDI) substrate for protein (Gain et al., 1994), carbohydrate (Bornsen et al., 1995; Jacobs and Dahlman, 2001), and DNA (Bai et al., 1994) detection. We use conductive polymer spray ionization (CPSI) as the representative spray MS method (Song et al., 2018) to investigate the desalting function of Nafion and its potential value in biofluid analysis. The Nafion solution will be coated onto the surface of a conductive polymer tip (as the sample loading and ionization substrate) to form a uniform membrane. The sulfonic acid group in the Nafion membrane is supposed to desalt the biofluid sample by exchanging its own proton with the sodium and potassium ion. An incubation period for this cation exchange can be synchronized with the biofluid drying process. Then, one droplet of solvent can be loaded onto the dried biofluid spot to carry the desalted components into the MS for data collection. Figure 1 displays this procedure.

## Results and Discussion

### Desalting Effect and Signal Enhancement

**FIGURE 2:**
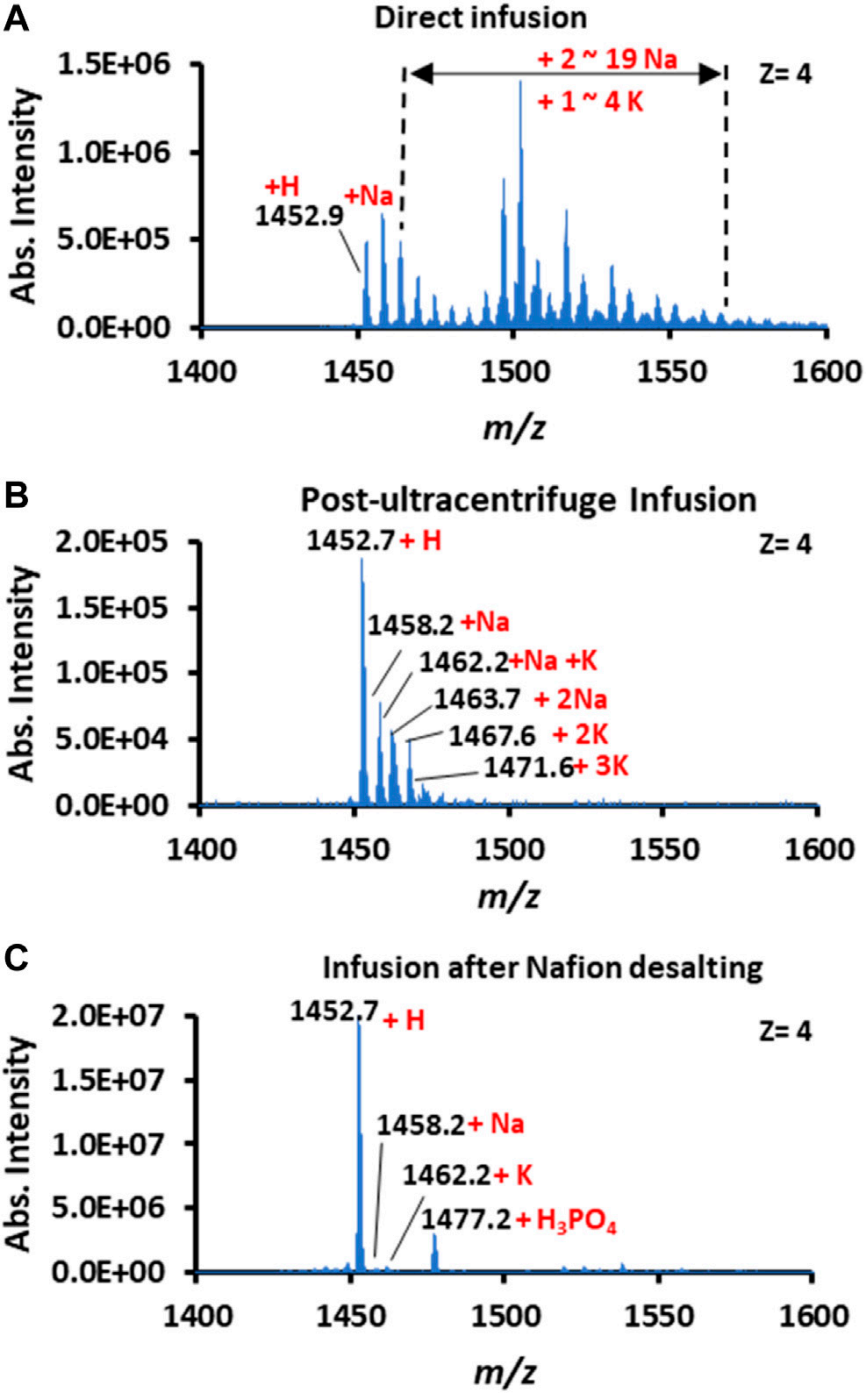
Mass spectra of insulin acquired by **(A)** direct infusion, **(B)** post-ultracentrifuge infusion, and **(C)** infusion after Nafion desalting.

Human recombinant insulin (5.8 kDa) was selected as the model protein to investigate Nafion’s role in spray MS analysis. An equal amount of insulin solution (250 μg/ml) was loaded onto the surface of a plain and a Nafion-coated conductive polymer tip, respectively. As a positive control, the same insulin solution was also pretreated with ultracentrifugation to remove native salts. As a result, the CPSI mass spectrum showed that multiply charged peak clusters were widely distributed with a range of *m*/*z* 1,450–1,600 (*z* = 4). These indicated that insulin molecules formed various adducts with varying amounts of proton or metal ions natively existing in solution. According to the delta *m*/*z* value between each peak cluster (△*m*/*z* = 5.5 for 1 Na^+^ and △*m*/*z* = 9.75 for 1 K^+^), the identity of each peak cluster’s adduct ion types can be assigned to sodium (predominant species) and potassium with the number of adduct ions varied from 1 to 19 for Na^+^ or 1 to 4 for K^+^ (Figure 2A).

Compared to the control, the CPSI mass spectra acquired from the ultracentrifuge purified and Nafion-incubated insulin became relatively clean. The insulin molecule after ultracentrifuge only carried 1 or 2 Na^+^ or K^+^ adducts (Figure 2B), whereas the insulin after Nafion incubation preserved the pure protonated peaks (*m*/*z* 1,452.7, Figure 2C). Although the ultracentrifuge process can gain a relatively pure protonated insulin mass spectrum, the intensity of insulin did not increase; this may be caused by the inevitable loss of insulin from dilution and absorption onto the ultracentrifuge filter. In contrast, the insulin signal after Nafion incubation was enhanced by a factor of approximately 40 (from 5.0E5 to 2.0E7). One reason for the signal enhancement is the suppression of multiple adduct ion types. Another reason for this enhancement is that *in situ* Nafion processing saves more target molecules from transfer loss. This result indicates Nafion’s desalting effect on removing the inorganic cation ions from biofluids.

### Investigation of the Salt Tolerance Amount

**FIGURE 3:**
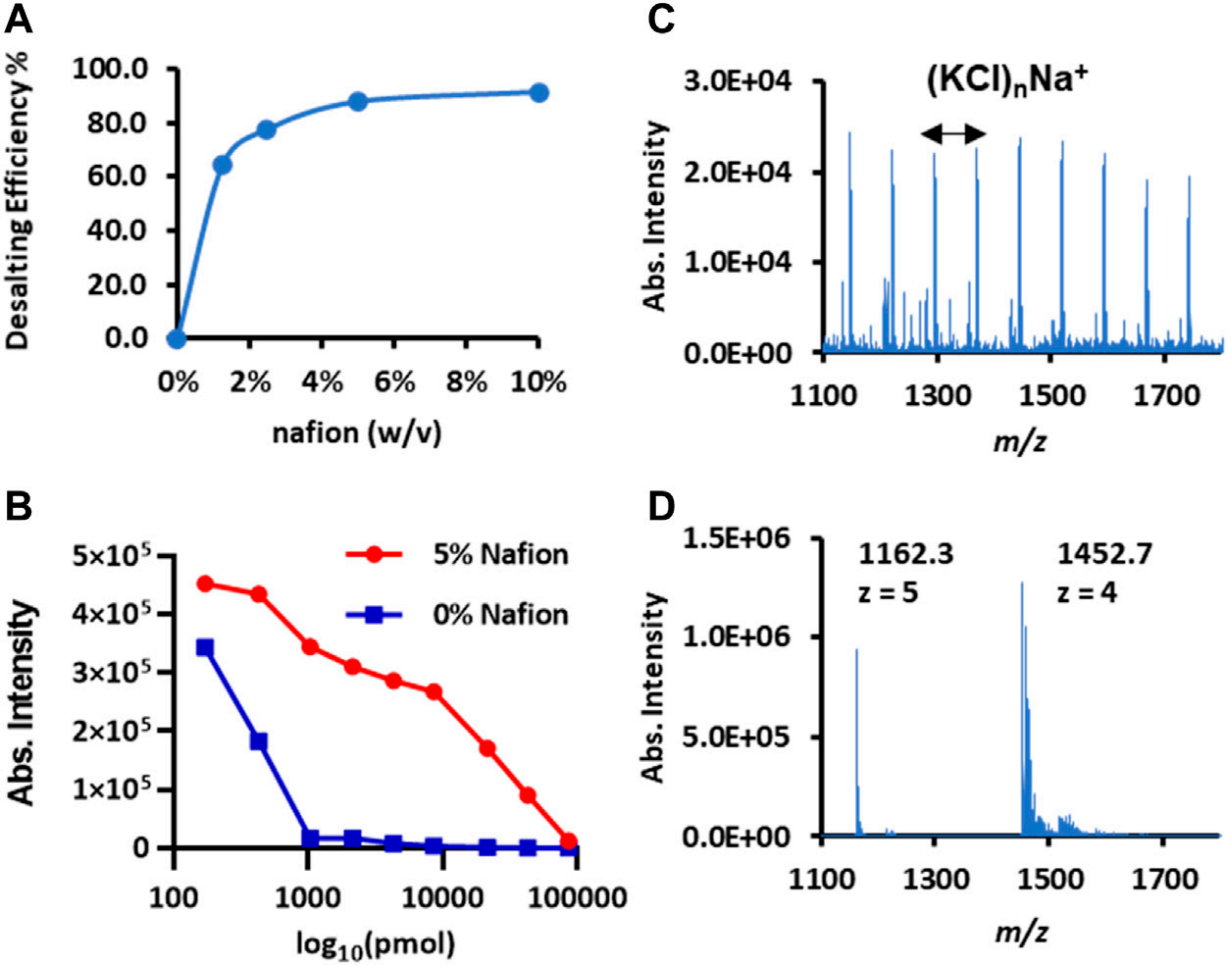
Desalting performance of Nafion coated on the conductive polymer surface for spray MS. **(A)** The curve of the desalting rate versus the concentrations of Nafion solution coated on the surface. Insulin (*m*/*z* 1,452.7, [M + 4H]^4+^) was used as the model protein. **(B)** The curves of protein intensity decline with the increased salt amount. Cytochrome *c* (*m*/*z* 1748.0, [M + 7]^7+^) was used as the model protein; the *x*-axis is the log_10_ value of the salt amount (NaCl). **(C)** The direct infusion mass spectrum of insulin (250 μg/ml) spiked in PBS buffer (pH 7.4). **(D)** The Nafion-desalting mass spectrum of the insulin solution same as **(C)**.

We continued investigating the influence of the Nafion coating amount on the desalting effect. Nafion solution with varying concentrations (1.0%, 2.5%, 5.0%, and 10%, w/v) was evenly coated onto the CPSI tip surface and dried for use. The intensity enhancement (IE%) and desalting efficiency (DE%) were introduced as two metrics to evaluate the desalting performance. The formulas are shown in Eqs 1, 2. $I_{H}$ and $I_{H}^{\prime }$ represent the normalized intensity of protonated ion before or after Nafion desalting, respectively. $I_{i}$ and $I_{i}^{\prime }$ represent the normalized intensity of sodium or potassium ion before or after Nafion desalting, respectively. The “*i*” denotes the number of adducted Na^+^ or K^+^ (*i* = 1, 2, 3, … *n*). As a result, we found that the DE% gradually increased with the Nafion coating amount and reached a plateau level of around 90% when its concentration reaches 5.0% (Figure 3A).

$$IE\%=100\times \frac{I_{H}^{\prime }-I_{H}}{I_{H}}$$ (1)

$$DE\%=\frac{\sum_{1}^{n}\left(i\;\times \;I_{i}\right)-\sum_{1}^{n}\left(i\;\times \;I_{i}^{\prime }\right)}{\sum_{1}^{n}\left(i\;\times \;I_{i}\right)}$$ (2)

The salt tolerance amount that the Nafion membrane could handle was also investigated after fixing the optimal Nafion coating amount. Cytochrome *c* (500 μg/ml) solution was mixed with an equal volume of sodium chloride at different concentrations. Then, these sample solutions (5 μl/each) were loaded onto a set of plain polymer tips and Nafion-coated tips for CPSI-MS detection. The protonated ion intensity change with the absolute salt amount was plotted for a better comparison. As shown in Figure 3B, the protonated ion (*m*/*z* 1,748, *z* = 7) intensity curve plotted from the Nafion-CPSI-MS test performed a relatively slow depletion compared to that from the plain CPSI-MS group. The ionization suppression for the insulin turned to almost 100% in the plain CPSI-MS group when the loaded salt amount reached 1,000 pmol. In contrast, the ion intensity could still be kept at a relatively high level for the Nafion-CPSI-MS group until the salt amount was higher than 10,000 pmol. Its intensity remained 10% of the original level when the salt amount was 100,000 pmol.

Biological fluids are usually rich in salts to maintain normal physiological homeostasis. Even for the *in vitro* cell culture, the media contained a high concentration of inorganic salts. Saline and PBS buffer are frequently used to prepare two simulative solutions. The former one contains 154 mM NaCl, and the latter one has a more complex composition PBS buffer (137 mM NaCl, 2.7 mM KCl, 8 mM Na_2_HPO_4_, and 2 mM KH_2_PO_4_). Given these facts and the maximum salt loading amount (approximately 100,000 pmol), we estimated that the maximum biofluid loading volume that a Nafion membrane can undertake was approximately 0.65 μl. If the salt concentration of a biofluid sample is even higher than the saline or PBS sample, a dilution process is necessary to lower the salt into an acceptable amount. We tested the insulin prepared in the PBS buffer solution. The salt cluster ion peaks [e.g., (KCl)_n_Na^+^] were predominant and overwhelmed the protein ions (Figure 3C). In contrast, Nafion-CPSI-MS can successfully detect the protein signal when diluting the insulin buffer solution by an equal volume of 50 mM ammonium acetate water solution (Figure 3D).

### Proteomic Profiling

**FIGURE 4:**
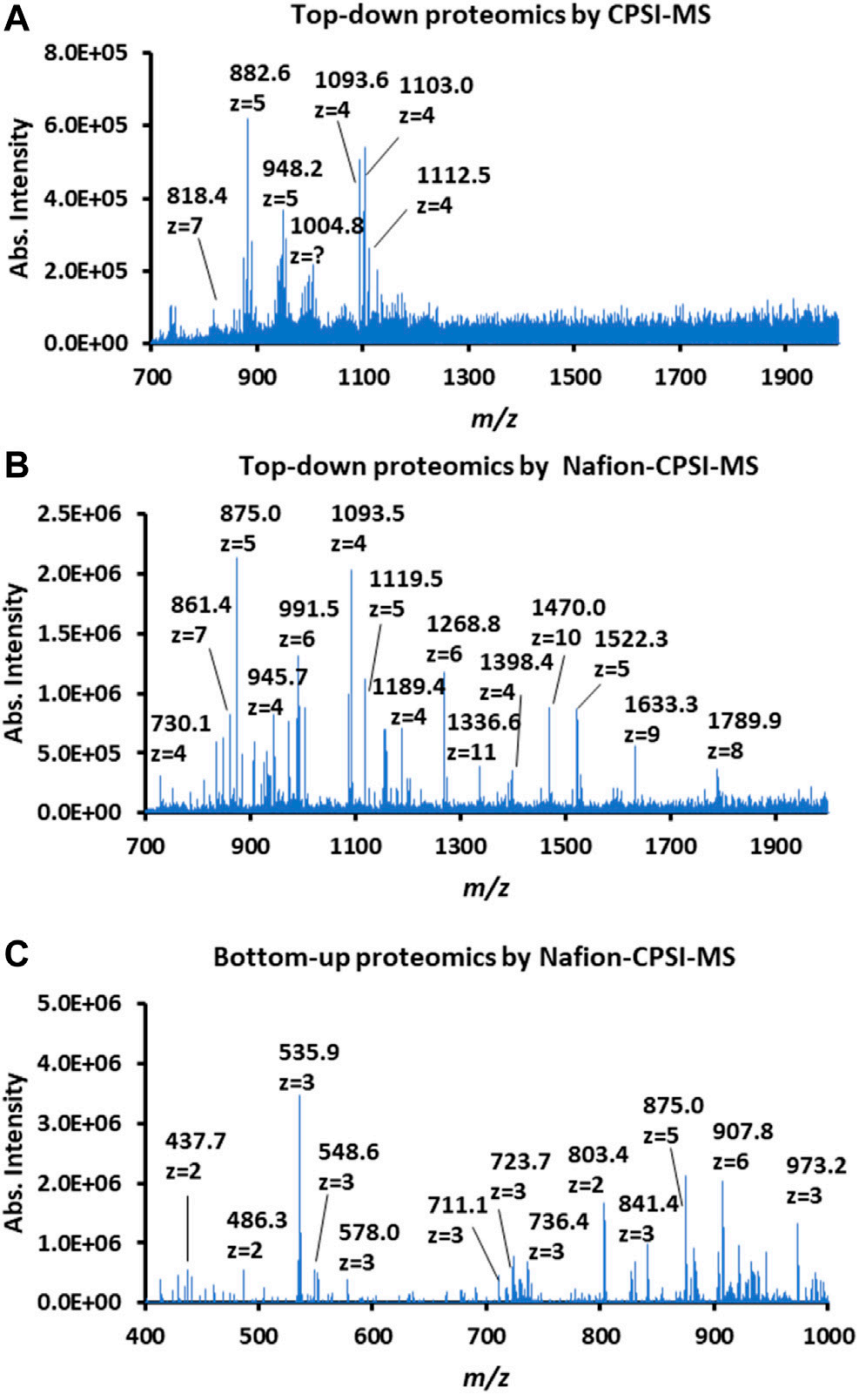
Performance of Nafion-coated CPSI-MS for proteomic profiling. **(A)** Proteomic profiling of 2-fold diluted saliva tested by a plain CPSI-MS. **(B)** Top-down proteomic profiling of 2-fold diluted saliva tested by Nafion-CPSI-MS. **(C)** Bottom-up proteomic profiling of 2-fold diluted saliva tested by Nafion-CPSI-MS.

We further tested the possibility of Nafion-CPSI-MS in salivary proteomic profiling. After ultracentrifugation, 1 μl of 2-fold diluted saliva was transferred to the Nafion membrane for further desalting. Additionally, a peptide mass fingerprint was also acquired after trypsin digestion. Compared to plain CPSI-MS (Figure 4A), the Nafion-CPSI-MS can collect more protein peaks with higher intensities (Figure 4B). There were at least 48 distinct proteins successfully detected with the molecular weight ranging from 1.4 to 14.7 kDa (Supplementary Table S1). In terms of the bottom-up proteomic profile, 116 specific peptides were detected with the molecular weight ranging from 0.8 to 1.3 kDa (Supplementary Table S2). We believe that these molecules might have the potential in characterizing certain oral diseases in further clinical or medical studies.

During the proteomics profiling study, we found that contact with the Nafion membrane surface has an influence on charge distributions of protein ions. Taking cytochrome *c* as the example, although the sodium adduct peaks can be effectively eliminated by incubating the solution with Nafion, the charge number of cytochrome *c* shifted from *z* = 7 to *z* = 8, and then from *z* = 9 to 19 (Supplementary Figure S1). This indicated that the negatively charged strong acid group may induce the conformational change of a protein. This phenomenon might become more obvious with the increase of a protein’s molecular weight and high-order structure complexity. It also means that the Nafion-desalting strategy is more suitable for the bottom-up peptide mass fingerprinting compared to native MS, which should be very careful about the high-order structure intactness (Leney and Heck, 2017).

### Nucleotides Profiling

**FIGURE 5:**
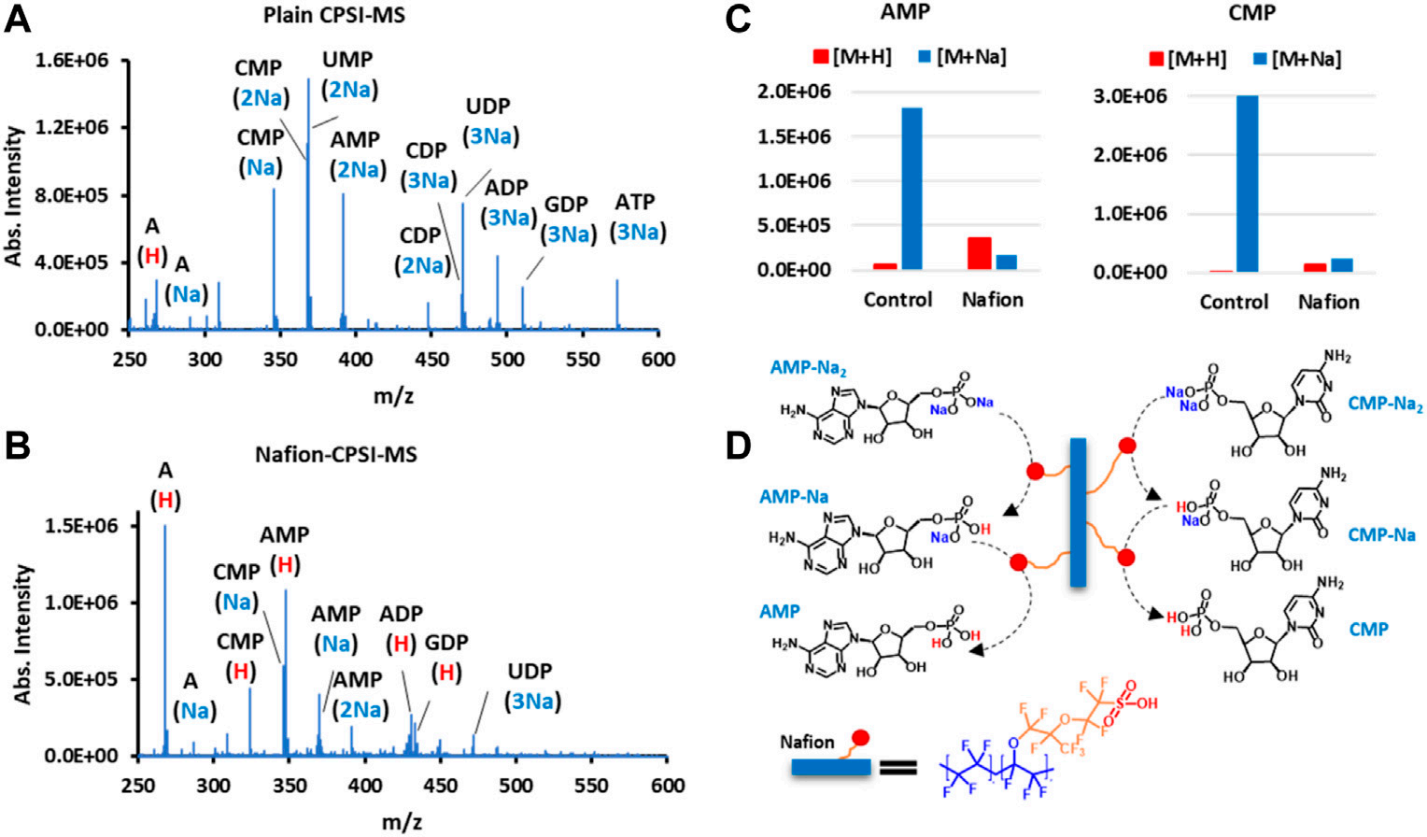
Nafion undergoes a proton exchange with the sodium ion binding in nucleotide molecules. **(A)** Nucleotide ions acquired by plain CPSI-MS. **(B)** Nucleotide ions acquired by Nafion-CPSI-MS. **(C)** Nafion’s desalting performances on two representative nucleotides, AMP and CMP. **(D)** Diagram of multi-step proton exchange happening in the interface of the Nafion membrane and nucleotides in biofluids.

Following up the proteomic profiling, the value of Nafion for facilitating spray MS in nucleotide detection was also evaluated. We prepared the solution composed of several nucleotide standards (Supplementary Table S3) for plain CPSI-MS and Nafion-CPSI-MS tests. The plain CPSI-MS revealed that the top 10 peaks all carried 1 to 3 sodium ions (Figure 5A). Because each phosphoric acid group can release protons and bind with more than one sodium ion under physiological pH, nucleotides are usually present as the mono-, di-, or triphosphate. However, the mass spectrum showed remarkable changes in profile after Nafion incubation. All sodiated peaks were obviously suppressed or even removed (Figure 5B). The protonated ions, in turn, were greatly increased such as cytidine monophosphate (CMP) and adenosine monophosphate (AMP) (Figure 5C).

We have already learned that the sulfonic acid group can exchange its protons with free sodium and potassium ions in a biofluid. From this section, it is also concluded that the Nafion’s sulfonic group can conduct a deeper, multi-step proton exchange with these cation ions binding within a metabolite, particularly those acidic ones such as phosphate (Figure 5D). Because sulfonic acid is quite strong (pKa: −6), this proton–cation ion exchange can be processed under a quite wide range of pH values, which suffices to cover most biological fluids.

### Normalizing Salt Variation and Linearity Improvement

**FIGURE 6:**
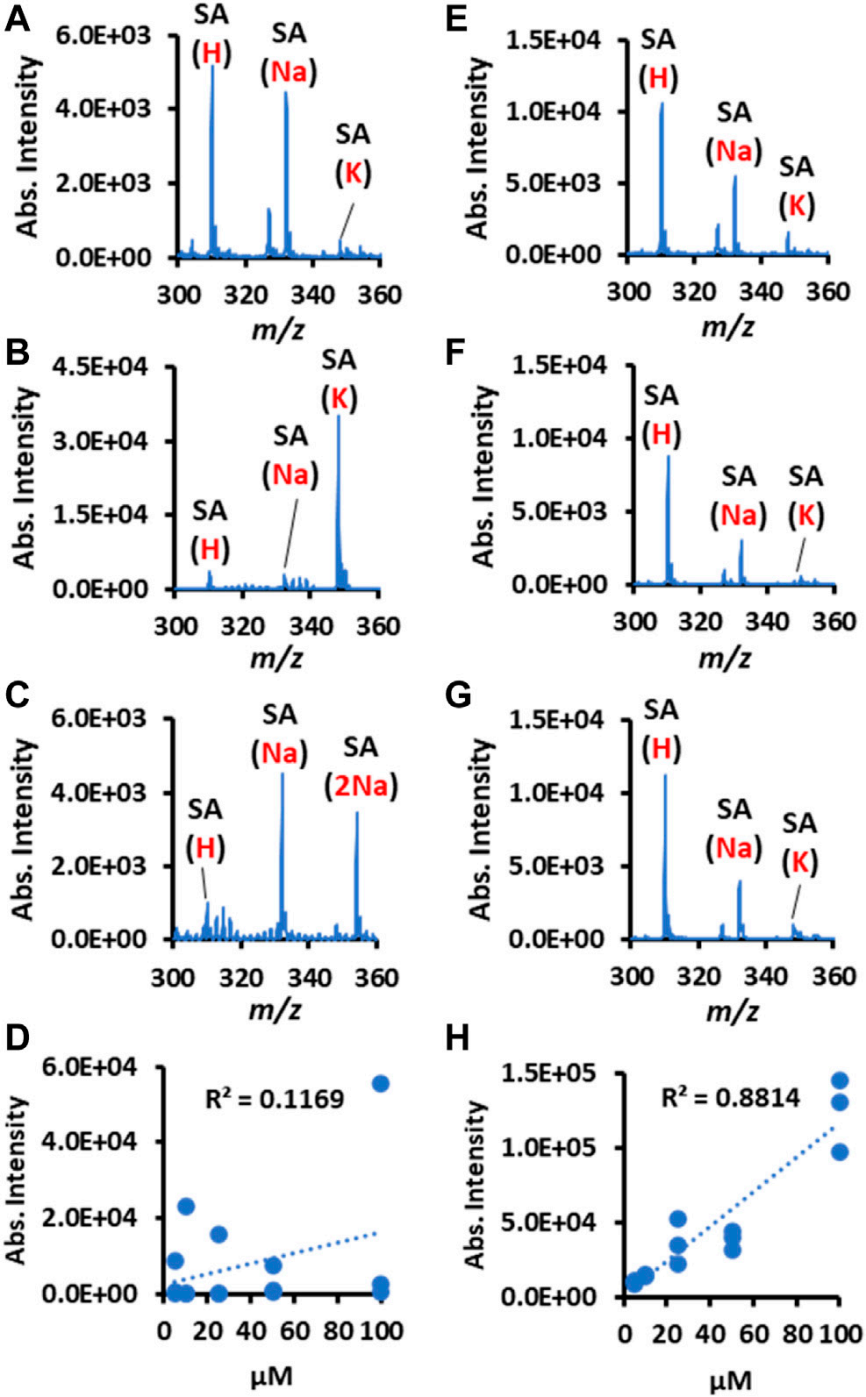
Nafion helps to normalize environmental salts variation and calibrate the linear response for quantitation. The left panel presents the plain CPSI-MS detection results for sialic acid (SA) ions detected from a solution spiked with **(A)** acetic acid, **(B)** potassium chloride, **(C)** sodium chloride, and **(D)** correlation between SA concentration and intensities. The right panel presents the Nafion-CPSI-MS detection results for SA ions detected from a solution spiked with **(E)** acetic acid, **(F)** potassium chloride, **(G)** sodium chloride, and **(H)** correlation between SA concentration and intensities.

The variation in biofluid salt concentration is a factor that is easily ignored during spray MS. However, it can cause an important influence on the relative abundance of different adduct ion types. As is shown in Figures 6A–C, the sialic acid (SA) at the same concentration has quite different adduct ion patterns because of their salt composition differences in the external environment. Consequently, the linear relationship between concentration-ion intensity becomes seriously distorted for qualitative comparison or accurate quantitation (Figure 6D). In contrast, the Nafion-CPSI-MS can chemically normalize the varied adduct ions into approximately the same level (Figures 6E–G). Therefore, the poor linearity was greatly improved with an increased linear response (Figure 6H).

### Universal Applicability

In this study, our CPSI-MS method (Song et al., 2018) was selected to illustrate the advantage of Nafion membrane for *in situ* desalting process. Given a maximum salt tolerance amount, the sample loading volume might be at a sub-microliter or even lower if a biofluid contains a very high salt concentration. However, it hinted to us that the Nafion-based spray MS might also have the potential for those trace volume biofluids such as sweat, tears, interstitial fluids, or even trace amounts of suspension cells (cytoplasm at a pL level). In terms of the solvent, these MS-compatible buffers are completely compatible with ammonium acetate, ammonium bicarbonate, and formic acid. Because Nafion will be more easily dissolved in organic solvents, it might have a higher chance of being exposed to the organic solvent after the dried fluid spot sample is completely consumed. Therefore, although the regular solvent for MS such as methanol and acetonitrile is compatible, a low volume percentage (less than 50%) is recommended, which is also suitable for the MS analysis of native proteins.

In terms of target species, the Nafion desalting process is demonstrated to be applicable for peptides, proteins, nucleotides, or other acidic metabolites such as carboxylate compounds. Additionally, it is worth noting that this *in situ* desalting material is supposed to be a universal solution for various alternative spray MS substrates such as for porous ones like paper (Damon, et al., 2016), or those hydrophobic materials such as organosiloxanes (Dulay and Zare, 2017; Dulay et al., 2021), polystyrene (Wang et al., 2017), carbon nanotubes (Song et al., 2020), and Teflon (Narayanan et al., 2020).

## Conclusion

It is demonstrated that Nafion coating a spray tip successfully integrates the desalting process with spray MS. The sulfonic acid group from the Nafion membrane exchanges protons with sodium and potassium ions both in the free form in biofluids and binding form contained in metabolites. This simple, *in situ* desalting process has several advantages over direct infusion analysis: (1) the ionization and sensitivity can be enhanced by removing the multiply adducted ion types and narrowing adduct ion distributions down to the proton-adduct one; (2) a metabolite’s intensity changes from environmental ion variations can be normalized for a more fairer qualitative comparison; and (3) the linear response between concentration and intensity is enhanced and the linear range is widened for quantitation. In general, this study also provides a proof-of-concept strategy that functional polymers can be used as the spray MS probe to conveniently implement various sample pretreatment procedures before direct infusion mass spectrometry analysis of biofluids.

## Materials and Methods

### Chemicals and Instrument

Methanol, ultrapure water, formic acid, sodium chloride, potassium chloride, and ammonium acetate were purchased from Fisher Chemical. Nafion perfluorinated resin solution (20 wt% in lower aliphatic alcohols and water), trypsin, insulin, cytochrome c, N-acetyl neuraminic acid, nucleotides, and nucleoside standards were all purchased from Sigma-Aldrich. Polymethyl methacrylate (PMMA, molecular weight within 30–90 kDa) and multi-walled carbon nanotube (MWCNT, ID 2–5 nm, OD < 8 nm, length 10–30 μm) were purchased from Adamas-beta Reagent Ltd. and J&K Scientific, respectively. Ultracentrifuge spin columns (cutoff molecular weight: 3.0 kDa) were purchased from Millipore. The LTQ Orbitrap Velos mass spectrometer (Thermo Scientific, San Jose, CA) was employed for recording the mass spectra data.

### Preparation of Nafion-Coated Conductive Polymer

Fabrication of the conductive polymer can be accessed from the previous report (Song et al., 2018). Briefly, PMMA particles (1.5 g) are spiked into 5.0 ml acetyl acetate and refluxed at 120°C until fully dissolved. MWCNT (150.0 mg) is added to another 5.0 ml acetyl acetate followed by ultrasonication until homogenously dispersed. Same volumes (1.5 ml) of MWCNT and PMMA solutions were then cast into a 10-ml beaker sealed with punctured parafilm. With the slow evaporation of the organic solvent, the MWCNT/PMMA composite was gradually re-molded into a uniform, black, polymer. The raw conductive polymer was then cut into triangular shapes (10 mm in height and 8 mm in base) and rinsed with water before use. The Nafion solution (20 wt%) was first diluted to 1.25%, 2.5%, 5%, and 10% for optimal concentration investigation. The diluted Nafion solution (2.0 μl) was then micropipetted onto the conductive polymer tip to form a thin membrane after full dryness.

### Sample Collection and Processing

Saliva was harvested from the first author himself under no external stimulus and tested immediately. For metabolic profiling, only 1 μl of raw saliva was transferred into a blank plastic tube and diluted with 9 μl of ultrapure water for use. Then, a microloader (Eppendorf™ Femtotips™) was employed to accurately transfer 0.5 μl of diluted saliva (the endogenous component amount was equivalent to 50 nl of raw saliva) onto the Nafion membrane coated on the conductive polymer tip to initiate the desalting process. For salivary proteomic profiling, 500 μl of saliva was first centrifuged at 3,000 rpm for 5 min. Then, a 200-μl supernatant was loaded three times (15 min/each time) onto a spin column for ultracentrifugation at 12,000 rpm. The residue saliva solution preserved on the spin column film was compensated with ammonium acetate (50 mM) solution to 400 μl (equivalent to a 2-fold dilution). Another 200-μl supernatant was spiked with trypsin powder (extracted from porcine pancreas, salt-free, lyophilized powder, 0.5 mg) for generating the peptide mass fingerprint. A 1-μl diluted saliva sample solution was transferred to the Nafion membrane for further desalting. No additional pretreatment was needed before on-surface Nafion desalting, which can also be completed during the biofluid drying process.

### Solution Preparation

Cytochrome *c* and insulin were dissolved with ultrapure water with final concentrations both at 50 μM. Different amounts of sodium chloride (4, 10, 20, 50, 100, 200, 500, 1,000, and 2,000 μg/ml) were spiked into an equal volume of cytochrome *c* or insulin solution for evaluating the desalting efficiency of Nafion-coated conductive polymer. In contrast, cytochrome *c* or insulin was also loaded into a spin column for ultracentrifuge (15,000 rpm, 15 min, three times). The sialic acid (SA) was first dissolved in ultrapure water as the stock solution (1.0 mM). Then, a series of diluted solutions were prepared with concentrations at 5, 10, 25, 50, and 200 μM, respectively. Then, 5 μl of sodium chloride (100 μM), potassium chloride (100 μM), or formic acid (0.5%) was externally spiked into the 50-μl serial diluted SA solutions for investigating the ionization suppression and efficiency, respectively.

### MS Data Acquisition

The general procedure was the same as the previously reported CPSI-MS approach (Song et al., 2018) after precoating the Nafion membrane as described above. Briefly, the 1-μl biological fluid or pure standard solution was first loaded onto the conductive polymer tip, which was set at a 10.0-mm distance away from the MS inlet. When the sample was evaporated to form a dried fluid spot, methanol–water (5:5, v/v, 3 μl) was used as extraction and spraying solvent. The ammonium acetate (50 mM) was also additionally spiked for protein sample analysis. Once the +5-kV high voltage is applied onto the Nafion-coated conductive polymer, the charged microdroplets spray will be generated and carry the extracted components into the mass spectrometer. The general full scan range was set at *m*/*z* 50–400 for metabolic profiling and *m*/*z* 200–2,000 for proteomic profiling under positive mode. Specific scan range depends on the analyte of interest. The MS capillary temperature was set at 300°C with the tube lens voltage set at 55 V. The automatic gain control was set at 3E6 with the maximum injection time set at 400 ms. The Xcalibur software (Thermo scientific) was first used for generating average mass spectra and converting raw files into cdf files. Each average mass spectrum was generated based on 20 continuous MS scans. The ion intensity was normalized with the total ion current (TIC) value.

## Data Availability

The original contributions presented in the study are included in the article/Supplementary Material. Further inquiries can be directed to the corresponding author.
