# Supplementary material for: Introducing Nafion for In Situ Desalting and Biofluid Profiling in Spray Mass Spectrometry
Source: Front Chem. 2022 Jan 25;9:807244. doi: 10.3389/fchem.2021.807244 (PMC8821663; doi:10.3389/fchem.2021.807244)
Supplement: Supplementary file 1 [file DataSheet1.docx]

**SUPPLEMENTARY INFORMATION**

**Introducing Nafion for *in situ* Desalting and Biofluid Profiling in Spray Mass Spectrometry**

Xiaowei Song^1,2^, Mohammad Mofidfar^2^, Richard N. Zare^2^*

1. Department of Chemistry, Fudan University, Shanghai, China. 200438
2. Department of Chemistry, Stanford University, Stanford, CA, USA. 94305

**Table of Contents**

| **Tables or Figures** | **Legend** | **Pages** |
| --- | --- | --- |
| Table S1 | List of salivary proteins successfully detected by Nafion-CPSI-MS in a top-down strategy | S2 |
| Table S2 | List of salivary peptides successfully detected by Nafion-CPSI-MS in a bottom-up strategy. | S3 |
| Table S3 | Ion intensities of nucleoside and nucleotides with and without Nafion desalting process. | S4-S5 |
| Figure S1 | Mass spectra of cytochrome *c* acquired from plain CPSI-MS and Nafion-CPSI-MS. | S6 |

**Table S1. List of salivary proteins successfully detected by Nafion-CPSI-MS in a top-down strategy.**

| **MW** | **m/z** | **z** | **MW** | **m/z** | **z** |
| --- | --- | --- | --- | --- | --- |
| 1469 | 735.5 | 2 | 6921.6 | 1154.6 | 6 |
| 2356.2 | 786.4 | 3 | 6922.3 | 989.9 | 7 |
| 2720.4 | 907.8 | 3 | 6922.5 | 1385.5 | 5 |
| 2916.4 | 730.1 | 4 | 6933.5 | 991.5 | 7 |
| 3778.8 | 945.7 | 4 | 6949.5 | 1390.9 | 5 |
| 4369.2 | 729.2 | 6 | 6949.6 | 993.8 | 7 |
| 4370 | 875 | 5 | 6949.8 | 1159.3 | 6 |
| 4370 | 1093.5 | 4 | 6950.4 | 869.8 | 8 |
| 4370.4 | 1093.6 | 4 | 6961.5 | 995.5 | 7 |
| 4408 | 1103 | 4 | 6986.7 | 999.1 | 7 |
| 4409 | 882.8 | 5 | 7606.4 | 951.8 | 8 |
| 4753.6 | 1189.4 | 4 | 7606.5 | 1522.3 | 5 |
| 5589 | 932.5 | 6 | 7606.8 | 1268.8 | 6 |
| 5589.6 | 1398.4 | 4 | 7607.5 | 1522.5 | 5 |
| 5592.5 | 1119.5 | 5 | 7607.6 | 1087.8 | 7 |
| 5628 | 939 | 6 | 7630 | 1091 | 7 |
| 5628 | 1126.6 | 5 | 7644.8 | 956.6 | 8 |
| 5683.3 | 812.9 | 7 | 7645.5 | 1530.1 | 5 |
| 5685 | 948.5 | 6 | 7645.8 | 1275.3 | 6 |
| 5685 | 1138 | 5 | 7676.4 | 1280.4 | 6 |
| 5826.8 | 1457.7 | 4 | 7683.9 | 1098.7 | 7 |
| 5840.8 | 835.4 | 7 | 7685.5 | 1538.1 | 5 |
| 5842 | 1169.4 | 5 | 7744.5 | 1549.9 | 5 |
| 5842.2 | 974.7 | 6 | 9592.8 | 1200.1 | 8 |
| 5842.4 | 731.3 | 8 | 9593.5 | 1371.5 | 7 |
| 5879.3 | 840.9 | 7 | 9594.6 | 1600.1 | 6 |
| 5943 | 991.5 | 6 | 9630.4 | 1204.8 | 8 |
| 5943 | 1189.6 | 5 | 9669.6 | 1209.7 | 8 |
| 5980.8 | 855.4 | 7 | 11514 | 960.5 | 12 |
| 5982 | 998 | 6 | 11514 | 1152.4 | 10 |
| 6022.8 | 861.4 | 7 | 11519.3 | 887.1 | 13 |
| 6023 | 1205.6 | 5 | 14311.2 | 1789.9 | 8 |
| 6023.2 | 753.9 | 8 | 14690 | 1470 | 10 |
| 6023.4 | 1004.9 | 6 | 14690.7 | 1633.3 | 9 |
| 6061.8 | 1011.3 | 6 | 14691.6 | 1336.6 | 11 |
| 6062 | 1213.4 | 5 |  |  |  |

**Table S2. List of salivary peptides successfully detected by Nafion-CPSI-MS in a bottom-up strategy.**

| **MW** | **m/z** | **z** | **MW** | **m/z** | **z** | **MW** | **m/z** | **z** |
| --- | --- | --- | --- | --- | --- | --- | --- | --- |
| 873.4 | 437.7 | 2 | 1340.6 | 671.3 | 2 | 2126 | 1064 | 2 |
| 880.2 | 441.1 | 2 | 1356.6 | 679.3 | 2 | 2131.2 | 711.4 | 3 |
| 895.4 | 448.7 | 2 | 1386.8 | 694.4 | 2 | 2146.2 | 716.4 | 3 |
| 913.6 | 457.8 | 2 | 1405.8 | 703.9 | 2 | 2159.1 | 720.7 | 3 |
| 918.6 | 460.3 | 2 | 1448.8 | 725.4 | 2 | 2167.2 | 723.4 | 3 |
| 934.8 | 468.4 | 2 | 1462.8 | 732.4 | 2 | 2282.1 | 761.7 | 3 |
| 947.6 | 474.8 | 2 | 1470.8 | 736.4 | 2 | 2299.2 | 767.4 | 3 |
| 960.6 | 481.3 | 2 | 1486.6 | 744.3 | 2 | 2313 | 772 | 3 |
| 970.6 | 486.3 | 2 | 1498.6 | 750.3 | 2 | 2320.2 | 774.4 | 3 |
| 985.4 | 493.7 | 2 | 1505.8 | 753.9 | 2 | 2339.1 | 780.7 | 3 |
| 988.6 | 495.3 | 2 | 1552.8 | 777.4 | 2 | 2339.2 | 1170.6 | 2 |
| 1001.6 | 501.8 | 2 | 1611.8 | 806.9 | 2 | 2356.2 | 786.4 | 3 |
| 1008.4 | 505.2 | 2 | 1650.8 | 826.4 | 2 | 2356.2 | 1179.1 | 2 |
| 1011.6 | 506.8 | 2 | 1663.8 | 832.9 | 2 | 2363.1 | 788.7 | 3 |
| 1022.8 | 512.4 | 2 | 1679.8 | 840.9 | 2 | 2377.2 | 793.4 | 3 |
| 1030.6 | 516.3 | 2 | 1685.8 | 843.9 | 2 | 2377.2 | 1189.6 | 2 |
| 1045.6 | 523.8 | 2 | 1730.8 | 866.4 | 2 | 2389.2 | 797.4 | 3 |
| 1053.4 | 527.7 | 2 | 1731 | 578 | 3 | 2399.1 | 800.7 | 3 |
| 1067.6 | 534.8 | 2 | 1766.8 | 884.4 | 2 | 2415 | 806 | 3 |
| 1075.6 | 538.8 | 2 | 1768.8 | 590.6 | 3 | 2431.2 | 811.4 | 3 |
| 1097.6 | 549.8 | 2 | 1804.8 | 903.4 | 2 | 2461.2 | 821.4 | 3 |
| 1103.6 | 552.8 | 2 | 1839.8 | 920.9 | 2 | 2497.2 | 833.4 | 3 |
| 1105.6 | 553.8 | 2 | 1856.8 | 929.4 | 2 | 2500.2 | 834.4 | 3 |
| 1110.8 | 556.4 | 2 | 1870 | 936 | 2 | 2689.5 | 897.5 | 3 |
| 1122.6 | 562.3 | 2 | 1878.8 | 940.4 | 2 | 2834.4 | 945.8 | 3 |
| 1125.6 | 563.8 | 2 | 1908 | 955 | 2 | 2855.4 | 952.8 | 3 |
| 1128.6 | 565.3 | 2 | 1956.9 | 653.3 | 3 | 2891.4 | 964.8 | 3 |
| 1141.6 | 571.8 | 2 | 1957 | 979.5 | 2 | 3271.6 | 818.9 | 4 |
| 1192.6 | 597.3 | 2 | 1995 | 666 | 3 | 3271.8 | 1091.6 | 3 |
| 1199.6 | 600.8 | 2 | 2016 | 673 | 3 | 3309.6 | 1104.2 | 3 |
| 1201.6 | 601.8 | 2 | 2017 | 1009.5 | 2 | 3310.8 | 828.7 | 4 |
| 1223.6 | 612.8 | 2 | 2028 | 1015 | 2 | 3499.8 | 584.3 | 6 |
| 1237.6 | 619.8 | 2 | 2055 | 686 | 3 | 3622.8 | 906.7 | 4 |
| 1245.6 | 623.8 | 2 | 2066.1 | 689.7 | 3 | 3633.6 | 1212.2 | 3 |
| 1261.6 | 631.8 | 2 | 2067 | 1034.5 | 2 | 4602 | 1151.5 | 4 |
| 1268.6 | 635.3 | 2 | 2069.1 | 690.7 | 3 | 4618 | 1155.5 | 4 |
| 1294.6 | 648.3 | 2 | 2088 | 697 | 3 | 4630.4 | 1158.6 | 4 |
| 1312.6 | 657.3 | 2 | 2097.9 | 700.3 | 3 | 6091 | 1219.2 | 5 |
| 1320.6 | 661.3 | 2 | 2105 | 1053.5 | 2 | 6315 | 1264 | 5 |
| 1334.8 | 668.4 | 2 | 2124.9 | 709.3 | 3 | 6353 | 1271.6 | 5 |

**Table S3. Ion intensities of nucleoside and nucleotides with and without Nafion desalting process.**

| **metabolites** | **Formula** | **adduct ion** | **m/z** | **No desalting** | **Nafion desalting** | **Intensity**  **Enhancement %** |
| --- | --- | --- | --- | --- | --- | --- |
| Adenosine | C10H13N5O4 | [M+H]+ | 268.1038 | 3.0E+05 | 1.5E+06 | 409% |
| Adenosine | C10H13N5O4 | [M+Na]+ | 290.0860 | 7.7E+04 | 1.5E+04 | -81% |
| AMP | C10H14N5O7P | [M+H]+ | 348.0703 | 6.2E+04 | 3.6E+05 | 479% |
| AMP | C10H14N5O7P | [M+Na]+ | 370.0520 | 1.9E+05 | 1.3E+05 | -31% |
| AMP | C10H14N5O7P | [M+2Na-H]+ | 392.0340 | 8.1E+05 | 1.7E+04 | -98% |
| UMP | C9H13N2O9P | [M+H]+ | 325.0431 | 8.0E+01 | 6.2E+03 | 7714% |
| UMP | C9H13N2O9P | [M+Na]+ | 347.0251 | 2.1E+04 | 3.9E+04 | 82% |
| UMP | C9H13N2O9P | [M+2Na-H]+ | 369.0066 | 1.5E+06 | 4.5E+04 | -97% |
| CMP | C9H14N3O8P | [M+H]+ | 324.0586 | 6.3E+03 | 1.5E+05 | 2207% |
| CMP | C9H14N3O8P | [M+Na]+ | 346.0408 | 8.3E+05 | 2.0E+05 | -77% |
| CMP | C9H14N3O8P | [M+2Na-H]+ | 368.0223 | 1.1E+06 | 2.2E+04 | -98% |
| GMP | C10H14N5O8P | [M+H]+ | 364.0653 | ND | 1.4E+04 | inf |
| GMP | C10H14N5O8P | [M+Na]+ | 386.0472 | 2.0E+03 | 1.4E+04 | 626% |
| GMP | C10H14N5O8P | [M+2Na-H]+ | 408.0291 | 6.1E+04 | 3.9E+03 | -94% |
| ADP | C10H15N5O10P2 | [M+H]+ | 428.0367 | ND | 2.7E+04 | inf |
| ADP | C10H15N5O10P2 | [M+Na]+ | 450.0186 | 2.0E+03 | 3.6E+04 | 1744% |
| ADP | C10H15N5O10P2 | [M+2Na-H]+ | 472.0005 | 1.0E+05 | 4.7E+04 | -55% |
| ADP | C10H15N5O10P2 | [M+3Na-2H]+ | 493.9825 | 4.4E+05 | 6.5E+03 | -99% |
| UDP | C9H14N2O12P2 | [M+H]+ | 405.0095 | ND | 1.7E+03 | inf |
| UDP | C9H14N2O12P2 | [M+Na]+ | 426.9914 | ND | 8.0E+03 | inf |
| UDP | C9H14N2O12P2 | [M+2Na-H]+ | 448.9734 | 1.8E+04 | 1.6E+04 | -15% |
| UDP | C9H14N2O12P2 | [M+3Na-2H]+ | 470.9549 | 7.5E+05 | 1.9E+04 | -98% |
| CDP | C9H15N3O11P2 | [M+H]+ | 404.0255 | ND | 7.5E+03 | inf |
| CDP | C9H15N3O11P2 | [M+Na]+ | 426.0074 | 2.5E+03 | 1.3E+04 | 414% |
| CDP | C9H15N3O11P2 | [M+2Na-H]+ | 447.9893 | 1.6E+05 | 1.9E+04 | -88% |
| CDP | C9H15N3O11P2 | [M+3Na-2H]+ | 469.9713 | 2.1E+05 | 3.3E+03 | -98% |
| GDP | C10H15N5O11P2 | [M+H]+ | 444.0316 | ND | 9.9E+03 | inf |
| GDP | C10H15N5O11P2 | [M+Na]+ | 466.0136 | ND | 1.3E+04 | inf |
| GDP | C10H15N5O11P2 | [M+2Na-H]+ | 487.9955 | 1.9E+04 | 9.4E+03 | -49% |
| GDP | C10H15N5O11P2 | [M+3Na-2H]+ | 509.9774 | 2.5E+05 | 5.3E+03 | -98% |

**Table S3. (continued)**

| **metabolites** | **Formula** | **adduct ion** | **m/z** | **No desalting** | **Nafion desalting** | **Intensity**  **Enhancement %** |
| --- | --- | --- | --- | --- | --- | --- |
| ATP | C10H16N5O13P3 | [M+H]+ | 508.0030 | ND | 5.1E+03 | inf |
| ATP | C10H16N5O13P3 | [M+Na]+ | 529.9850 | ND | 8.1E+03 | inf |
| ATP | C10H16N5O13P3 | [M+2Na-H]+ | 551.9669 | 1.1E+04 | 1.3E+03 | -88% |
| ATP | C10H16N5O13P3 | [M+3Na-2H]+ | 573.9489 | 3.6E+04 | 6.8E+03 | -81% |
| UTP | C9H15N2O15P3 | [M+H]+ | 484.9758 | ND | 6.1E+02 | +inf |
| UTP | C9H15N2O15P3 | [M+Na]+ | 506.9577 | ND | 1.9E+03 | +inf |
| UTP | C9H15N2O15P3 | [M+2Na-H]+ | 528.9397 | ND | 3.6E+03 | +inf |
| UTP | C9H15N2O15P3 | [M+3Na-2H]+ | 550.9216 | 1.4E+04 | 5.2E+03 | -61% |
| CTP | C9H16N3O14P3 | [M+H]+ | 483.9918 | ND | 7.2E+02 | +inf |
| CTP | C9H16N3O14P3 | [M+Na]+ | 505.9737 | ND | 1.8E+02 | +inf |
| CTP | C9H16N3O14P3 | [M+2Na-H]+ | 527.9557 | ND | 6.8E+01 | +inf |
| CTP | C9H16N3O14P3 | [M+3Na-2H]+ | 549.9376 | 2.4E+03 | 0.0E+00 | -100% |
| GTP | C10H16N5O14P3 | [M+H]+ | 484.9758 | ND | 6.1E+02 | +inf |
| GTP | C10H16N5O14P3 | [M+Na]+ | 506.9577 | ND | 1.9E+03 | +inf |
| GTP | C10H16N5O14P3 | [M+2Na-H]+ | 528.9397 | ND | 3.6E+03 | +inf |
| GTP | C10H16N5O14P3 | [M+3Na-2H]+ | 589.9438 | 3.3E+02 | 2.9E+02 | -13% |


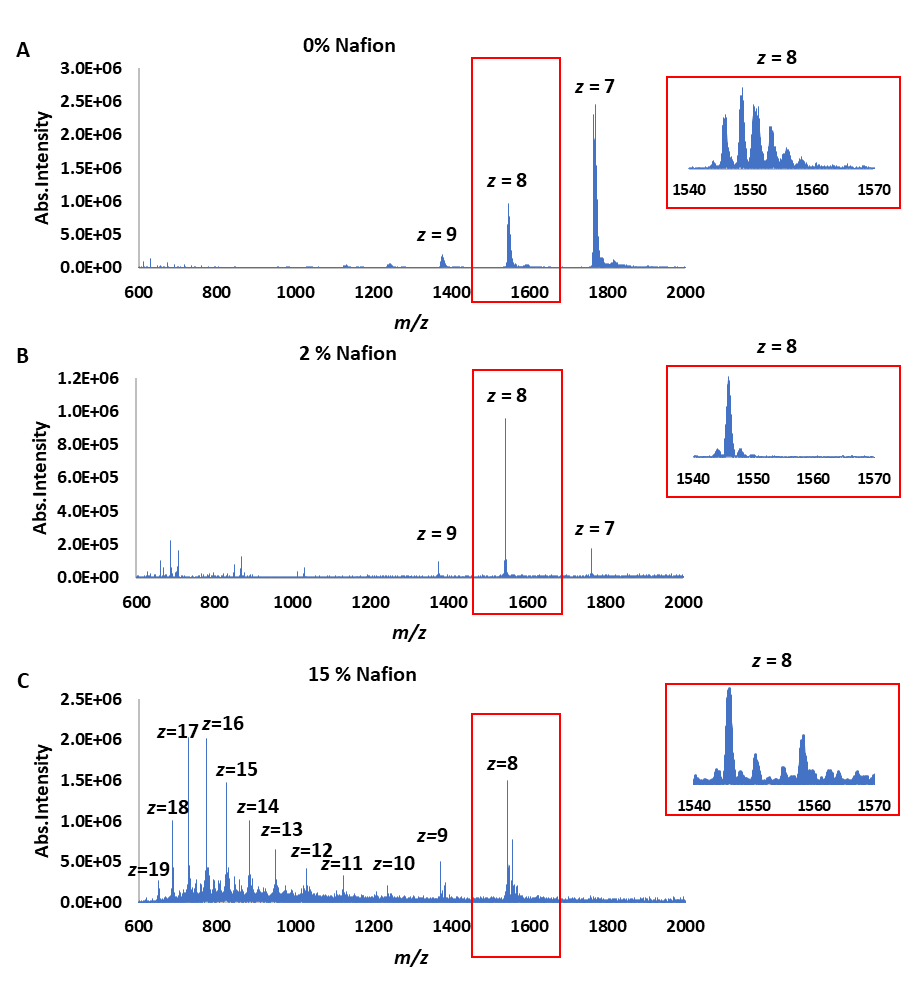


**Figure S1**. Mass spectra of cytochrome *c* acquired from plain CPSI-MS and Nafion-CPSI-MS. (A) no Nafion was coated onto the CPSI tip; (B) 2 % Nafion was coated onto the CPSI tip; (C) 15 % Nafion was coated onto the CPSI tip.
